# Association of Cognitive Function with Mortality and Cardiovascular Risk

**DOI:** 10.1002/alz.089353

**Published:** 2025-01-09

**Authors:** Jesus D Melgarejo, Sokratis Charisis, Dhrumil Patil, Kristina P. Vatcheva, Silvia Mejia‐Arango, Luis Javier Mena, Ney Alliey‐Rodriguez, Claudia L Satizabal, Carlos A Chavez, Ciro Gaona, Egle Silva, Gustavo Calmon, Joseph H. Lee, Joseph D. Terwilliger, Jose Gutierrez, Sudha Seshadri, Gladys E. Maestre

**Affiliations:** ^1^ Institute of Neuroscience, Harlingen, TX USA; ^2^ South Texas Alzheimer’s Disease Research Center, Harlingen, TX USA; ^3^ Glenn Biggs Institute for Alzheimer’s & Neurodegenerative Diseases, University of Texas Health Sciences Center at San Antonio, San Antonio, TX USA; ^4^ Beth Israel Deaconess Medical Centre, Harvard Medical School, Boston, MA USA; ^5^ School of Mathematical and Statistical Science, UTRGV, Brownsville, TX USA; ^6^ Institute of Neuroscience at the University of Texas Rio Grande Valley, Harlingen, TX USA; ^7^ Polytechnic University of Sinaloa, Maztlan, Sinaloa Mexico; ^8^ The University of Texas Rio Grande Valley School of Medicine, Harlingen, TX USA; ^9^ RGV Alzheimers Center (AD‐RCMAR), Brownsville, TX USA; ^10^ Glenn Biggs Institute for Alzheimer’s & Neurodegenerative Diseases, University of Texas Health Science Center at San Antonio, San Antonio, TX USA; ^11^ Laboratory of Neuroscience, University of Zulia, Maracaibo Venezuela (Bolivarian Republic of); ^12^ Laboratory of Ambulatory Recordings, Cardiovascular Institute (IECLUZ), University of Zulia, Maracaibo Venezuela (Bolivarian Republic of); ^13^ Columbia University Irving Medical Center, New York, NY USA; ^14^ Division of Medical Genetics, New York State Psychiatric Institute, New York, NY USA; ^15^ Sergievsky Center & Department of Epidemiology, Columbia University Medical Center, New York, NY USA; ^16^ Division of Public Health Genomics, National Institute for Health and Welfare, Helsinki Finland; ^17^ Vagelos College of Physicians and Surgeons, Columbia University, New York, NY USA; ^18^ Glenn Biggs Institute for Alzheimer’s & Neurodegenerative Diseases, University of Texas Health Science Center, San Antonio, TX USA; ^19^ South Texas Alzheimer’s Disease Research Center, San Antonio, TX USA; ^20^ The University of Texas Rio Grande Valley School of Medicine, Brownsville, TX USA

## Abstract

**Background:**

Cognitive impairment is associated with mortality. However, evidence using population‐based studies is scarce, is limited to one cognitive assessment, and lacks the inclusion of non‐fatal adverse health outcomes. Therefore, we aimed this study to (i) investigate the association of cognitive function with mortality and cardiovascular risk and (ii) determine whether cognitive tests improve the risk‐stratification of endpoints beyond conventional risk factors.

**Methods:**

We analyzed 946 participants aged ≥55y from a population‐based study who underwent cognitive function and were followed for fatal and nonfatal endpoints between 2001‐2016. Conventional risk factors included age, sex, education, body mass index, smoking, alcohol, antihypertensive treatment, office systolic and diastolic blood pressure, serum total cholesterol, diabetes mellitus, and previous history of cardiovascular diseases. Cognitive function examination included the mini‐mental state examination (MMSE) and selective reminder tests ([SRT], total retrieval, long‐term retrieval, and delayed recall). Our primary endpoint was total mortality and major adverse cardiovascular endpoints (MACE). Statistics included Cox proportional models and the generalized *R* ^2^ statistic to test model fit.

**Results:**

The mean age was 66.3±7.5 years old and 640 (67.6%) were women. During a median follow‐up of 8.5 years, 267 participants died and 187 experienced MACE. The incidence of mortality and MACE per quartiles of MMSE and SRT ranged from 13.1 (95% confidence interval [CI], 8.7‐18.9) to 23.8 (95% CI, 18.3‐30.4) among participants with the highest quartile and from 28.8 (95% CI, 14.9‐47.7) to 43.8 (26.3‐66.2) among participants with the lowest quartile. A lower MMSE score was associated with a higher risk of mortality (hazard ratios [HR] per each ‐4.3 lower MMSE, 1.23; 95% CI, 1.02‐1.48) and MACE (HR, 1.27; 95% CI, 1.09‐1.47). A lower SRT‐delayed recall score was associated with 1.19 (95% CI, 1.04‐1.37) and 1.24 (95% CI, 1.04‐1.46) higher folds of mortality and MACE endpoints. On top of conventional risk factors, the inclusions of MMSE and SRT improved the model performance (*R* ^2^ ranged from 0.51% to 1.65%; *P*≤0.028).

**Conclusions:**

In this population‐based study, cognitive impairment is associated with mortality and CV risk. Given the aging global population, the assessment of cognitive function might help reduce the risk of major preventable health outcomes.

